# Effectiveness of intraoperative cell salvage combined with a modified leucocyte depletion filter in metastatic spine tumour surgery

**DOI:** 10.1186/s12871-022-01743-0

**Published:** 2022-07-12

**Authors:** Ya-nan Zong, Chuan-ya Xu, Yue-qing Gong, Xiao-qing Zhang, Hong Zeng, Chang Liu, Bin Zhang, Li-xiang Xue, Xiang-yang Guo, Feng Wei, Yi Li

**Affiliations:** 1 Department of Anesthesiology, Peking University Third Hospital, No.49 Huayuanbei Road, Beijing, 100191 People’s Republic of China; 2 Biobank, Peking University Third Hospital, No.49 Huayuanbei Road, Beijing, China; 3 Department of Orthopaedic, Peking University Third Hospital, No.49 Huayuanbei Road, Beijing, 100191 People’s Republic of China

**Keywords:** Intraoperative cell salvage, Modified leucocyte depletion filter, Regular leucocyte depletion filter, Metastatic spine tumour surgery

## Abstract

**Background:**

To compare the effectiveness of intraoperative cell salvage (IOCS) combined with a modified leucocyte depletion filter (MLDF) with IOCS combined with a regular leucocyte depletion filter (RLDF) in eliminating tumour cells from blood salvage during metastatic spine tumour surgery (MSTS).

**Methods:**

Patients with a known primary epithelial tumour who underwent MSTS were recruited for this study. Blood samples were collected in 5 stages: from the patients’ vein before anaesthesia induction (S1), from the operative field at the time of maximum tumour manipulation (S2), and from the operative blood after IOCS processing (S3) and after IOCS+RLDF (S4) and IOCS+MLDF (S5) processing. The polyploids of tumour cells in the blood samples were collected and counted with immunomagnetic separation enrichment and fluorescence in situ hybridization.

**Results:**

We recruited 20 patients. Tumour cells were detected in 14 patients (70%) in S1, 16 patients (80%) in S2, 13 patients (65%) in S3, and 12 patients (60%) in S4. MLDF was added in 8 patients. Tumour cells were detected in only 1 of 8 patients in S5 (12.5%). There were significantly fewer tumour cells in the samples collected after MLDF processing (S5) than in the samples collected after RLDF (S4) and around the tumour (S2) (*P* = 0.016 and *P* = 0.039, respectively). Although no significant difference was observed between S4 and S1, a downward trend was observed after IOCS+RLDF processing.

**Conclusions:**

Tumour cells could be removed by IOCS combined with RLDF from blood salvaged during MSTS, but residual tumour cells remained. The findings support the notion that MLDF eliminates tumour cells more effectively than RLDF. Hence, this technique can be applied to MSTS.

**Trial registration:**

ChiCTR1800016162 Chinese Clinical Trial Registry.

## Background

Blood loss in spinal surgery, resulting from tumour hypervascularity, dilated epidural venous plexus, soft tissue paraspinal blood vessels, and even uninvolved bone, is still a major problem, especially in metastatic spine tumour surgery (MSTS) [1]. Currently, blood loss occurring in patients undergoing major tumour surgery is mainly replenished by allogeneic blood transfusion (ABT) [2]. However, there is increasing awareness of the fact that patients undergoing tumour surgeries are more susceptible to the adverse effects of ABT, such as transfusion-related infection, tumour growth promotion secondary to immunosuppression, and other transfusion reactions (e.g., allergic reactions, acute and delayed haemolytic reactions, and graft-versus-host disease) [3]. Moreover, ABT has been found to prolong hospital stays and increase medical costs compared to intraoperative cell salvage (IOCS) [4].

Intraoperative, salvaged, autologous blood transfusions carried out with autotransfusion devices are commonly used for cardiovascular surgery. They also enable the treatment of massive haemorrhage in musculoskeletal and gynaecologic surgeries to prevent potential complications of homologous blood transfusions. However, in oncologic surgery, transfusion of salvaged blood may cause haematogenous metastasis and dissemination of malignant tumour cells. Investigators have reported that blood irradiation or filtration using a leucocyte depletion filter (LDF) can prevent contamination with malignant tumour cells. Intraoperative autotransfusion combined with blood irradiation or LDF could be a promising technique for the treatment of profuse haemorrhage in oncologic surgery [5]. Prospective studies have shown that IOCS alone was successful in removing tumour cells in nearly 90% of the samples and that the combination of IOCS and LDF was more effective in removing tumour cells from blood salvaged during MSTS [6, 7]. However, this technique is still in its infancy in MSTS; thus, we conducted this study. Considering that the tumour cells in the blood are of different size due to different pathologic types of primary tumours, filters of two pore sizes were used in the experiment, regular LDF (RLDF, 40 μm) and modified LDF (MLDF, 18 μm) [8]. MLDF is an improved device compared with RLDF and is used for research purposes only at present. Although there have been several reports of RLDF and IOCS for MSTS, the study of MLDF reports is still in the exploratory stage. Currently, few studies have reported the application of combined IOCS and MLDF in MSTS. The purpose of this study was to analyse the ability of IOCS-MLDF to eliminate tumour cells and to evaluate the safety of autotransfusion based on laboratory test results in patients with MSTS.

## Methods

### Study design and study population

We recruited patients with metastatic spinal tumours who underwent spinal surgeries between May 2018 and May 2019 at our university hospital. The exclusion criteria were the presence of haematological or infectious diseases and not consenting to participate in the study. This study was approved by the ethics committee of Peking University Third Hospital with approval number LM2018020. The patients provided written consent. All methods were carried out in accordance with Declaration of Helsinki. Patient demographic information was recorded.

### Sample collection

The suction tube and blood reservoir of the IOCS machine (Cell Saver 5+; Haemonetics Corporation, Braintree, MA, USA) were rinsed and pre-filled with 200 mL of anticoagulant saline (heparin saline, 30 IU/mL). The negative pressure of the suction device was set at 120–150 mmHg. The anticoagulant drip rate was adjusted to approximately 100 drops/minute, and the flow rate was adjusted according to the amount and speed of the recovered blood. All intraoperative shed blood was recovered from the skin incision to remove the tumour. The recovered blood was anticoagulated and washed with sterilised saline (2000 mL for 125 mL of red blood cells). Only salvaged blood with a haematocrit of 30–60% and volume greater than 100 mL was eligible for use.

Two types of leucocyte reduction filters (RLDF and MLDF) were used in this experiment. The RLDF had a bore diameter of 40 μm (SB; Haemonetics Corporation). The MLDF had a bore diameter of 18 μm (Separator Haemo-Technology Beijing Co Ltd., Beijing, China). In the initial experiment, 12 patients were recruited with IOCS+RLDF, and it was found 5 patients (42%) still had residual tumour cells detected at S4 stage. In order to discover more effective methods, we consulted the literature and improved RLDF with MLDF, which had a smaller pore size. In the subsequent experiment, 8 patients were recruited and the blood was filtrated after IOCS process with RLDF and MLDF. Overall, 20 patients had IOCS+RLDF, in which 8 cases were treated additionally with IOCS+MLDF. Blood products treated with IOCS-LDF were used only for research purposes and not transfused to patients.

**Fig. 1:**
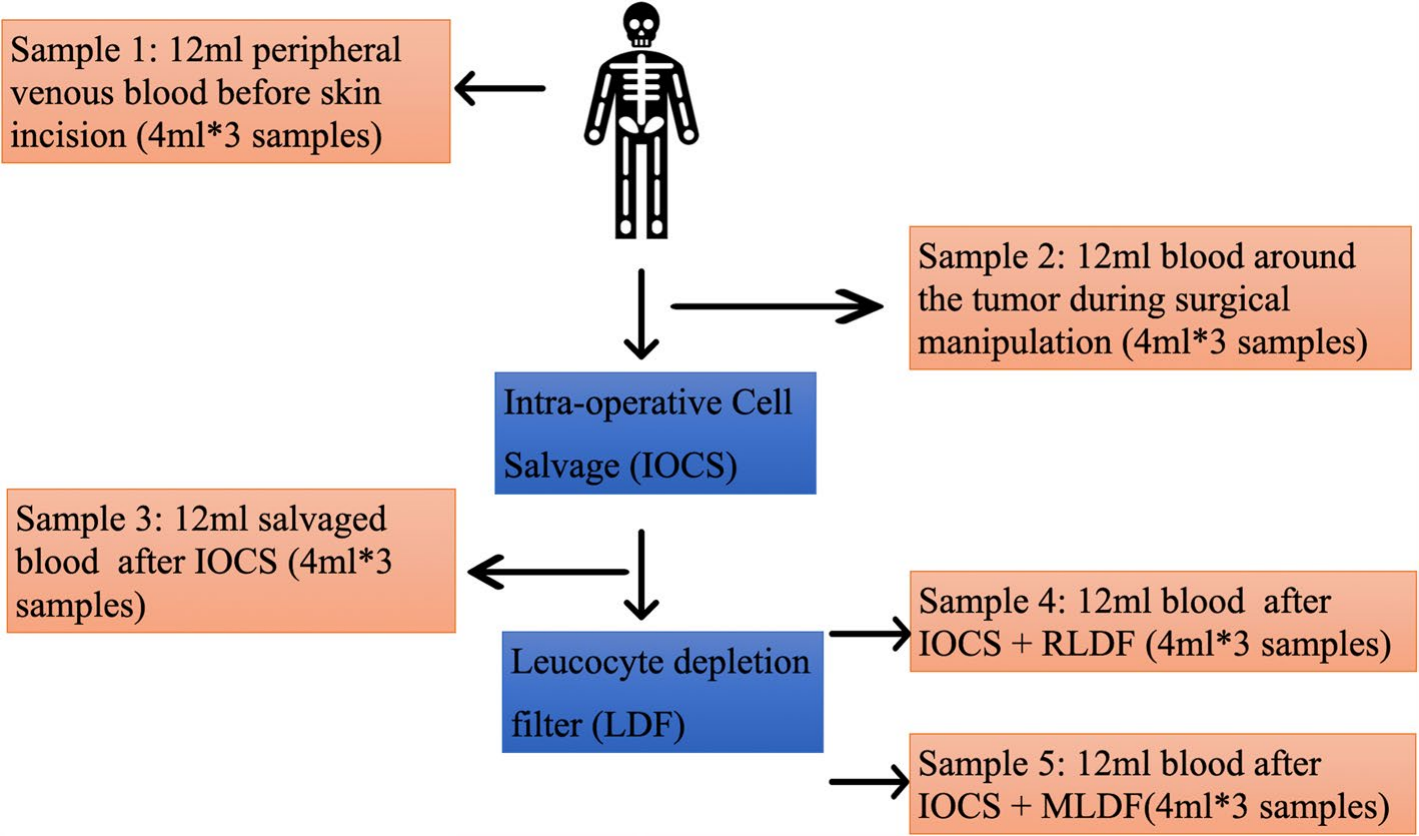
Stages of blood samples collection during surgery. There are 5 stages for blood sample collection. S1, peripheral venous blood from peripheral vein before skin incision; S2, blood sampled from the operative field at the time of maximum tumour manipulation; S3, IOCS blood after washing and before LDF filtering; S4, blood sample after IOCS+RLDF filtration; and S5, blood sample after IOCS+MLDF filtration. 12 ml blood sample was collected which comprised three separate 4 ml sample taken at slightly different time points to avoid sampling error

Blood samples were collected in 5 stages: S1, blood collected from the peripheral vein before the skin incision; S2, blood collected from the operative field at the time of maximum tumour manipulation; S3, IOCS blood collected after washing and before filtration using the LDF; S4, blood collected after filtration using IOCS+RLDF; and S5, blood collected after filtration using IOCS+MLDF. Twelve millilitres of blood were collected at each stage, which comprised 3 separate 4-mL samples collected at slightly different time points to avoid sampling error (Fig. 1). And the 3 samples taken at each stage were then pooled for laboratory analysis. All blood samples were stored in ethylenediaminetetraacetic acid-coated vacutainer tubes (Becton–Dickinson, Franklin Lakes, NJ, USA), and the tubes were stored at 4 °C.

### Laboratory methods

The polyploids of tumour cells in blood samples were collected and counted using immunomagnetic separation enrichment and fluorescence in situ hybridization (FISH). Tumour cells from blood samples were enriched using a human peripheral blood leucocyte removal kit (Cyttel®, Jiangsu, China). After centrifugation of blood samples, the red blood cells were lysed and the white blood cells were removed using CD45 immunomagnetic beads. CD45 expression was detected in the remaining cells by immunofluorescence staining to further exclude the remaining leucocytes. Probes were utilized to identify centromeres of chromosomes 8 and 17 or 7. FISH was used to determine the number of chromosomes. When the number of chromosomes 8, 17, or 7 exceeded 2, the cell was classified as aneuploid and determined to be a tumour cell. Chromosome enumeration probe 8 (CEP 8) was utilized for the detection of samples from various cancer types [9]. At the same time, chromosome 7 (CEP 7) was counted in renal [10], colon [11], oesophageal [12], and prostate [13] cancer cells, whereas chromosome 17 (CEP 17) was counted in endometrial and breast [14] cancer cells. The results were examined and judged by 2 independent pathologists.

### Statistical analysis

All data were analysed using SPSS 24.0 (IBM Corporation, Armonk, NY, USA.). Measurement data conforming to the normal distribution and homogeneity of variances are expressed as the mean ± standard deviation. Non-normally distributed data are expressed as the median (interquartile range). Within-group comparisons were performed using Wilcoxon analysis. *P* < 0.05 was considered statistically significant.

## Results

### Baseline and procedural characteristics

Between May 2018 and May 2019, 24 patients with metastatic spinal cord compression were enrolled. The IOCS-LDF device was used during surgery. Four patients were excluded because of insufficient blood samples and presence of osteosarcoma as detected on postoperative pathology. Surgical sites included the cervical vertebra (5 cases), cervicothoracic vertebra (1 case), thoracic vertebra (10 cases), and lumbar vertebra (4 cases). The average age of the 20 patients (12 men and 8 women) was 57.55 ± 9.71 years. And none of them had preoperative segment vascular embolism. All 20 patients underwent MSTS (intralesional resection), of which 11 cases were decompression and 9 cases were debulking. The surgical duration was 138.4 ± 47.06 minutes. The average volumes of blood loss, red blood cell transfusion, and autologous blood recovery were 392.5 ± 270.61, 280 ± 293.08, and 196.25 ± 135 mL, respectively (Table 1).

**Table 1:** Patient characteristics, surgical management, and numbers of tumour cell per 4 ml blood samples

Patient no.	Age / years	Gender	ASA status	Primary tumour	Location of the tumour	Procedure	Blood loss /ml	Blood transfusion / ml	Duration of surgery / min	Survival time / month	New metastasis / month	S 1	S 2	S 3	S 4	S5
1	60	Male	II	Lung	Cervical vertebra	Decompre-ssion	200	0	102	3	3	0	0	3	1	
2	78	Female	II	Lung	Cervical vertebra	Decompre-ssion	200	400	178	27	Unknown	0	2	3	5	
3	53	Female	I	Breast	Cervical vertebra	Decompre-ssion	150	0	74	Survival (> 47)	None	2	1	0	0	
4	44	Female	I	Breast	Lumbar vertebra	Decompre-ssion	600	0	124	30	None	2	2	1	0	
5	62	Male	I	Thyroid	Lumbar vertebra	Debulking	500	400	230	Survival (> 42)	21	6	6	2	0	
6	59	Female	II	Thyroid	Thoracic vertebra	Decompre-ssion	200	0	90	36	None	7	0	0	0	
7	63	Male	II	Colon	Lumbar vertebra	Debulking	200	0	156	12	6	8	34	1	2	
8	44	Female	II	Colon	Thoracic vertebra	Debulking	300	800	96	10	3	3	1	0	1	
9	63	Male	II	Esopha-gus	Cervical vertebra	Debulking	400	800	110	4	4	1	2	0	0	
10	69	Male	II	Esopha-gus	Thoracic vertebra	Decompre-ssion	200	400	87	16	Unknown	3	2	1	1	
11	50	Male	III	Rectum	Thoracic vertebra	Decompre-ssion	100	0	106	6	Unknown	1	2	4	0	
12	42	Female	II	Endome-trium	Cervicothoracic vertebra	Decompre-ssion	600	0	137	Unknown	Unknown	3	1	0	0	
13	61	Male	II	Ampulla	Lumbar vertebra	Debulking	800	400	121	Unknown	Unknown	1	1	2	1	0
14	44	Female	II	Breast	Cervical vertebra	Decompre-ssion	200	400	211	Survival (> 38)	None	2	1	1	2	0
15	48	Male	I	Nasopha-rynx	Thoracic vertebra	Debulking	1000	800	201	Survival (> 41)	None	2	0	0	0	0
16	66	Male	II	Renal	Thoracic vertebra	Debulking	800	400	111	21	Unknown	0	2	3	2	0
17	56	Male	II	Lung	Thoracic vertebra	Debulking	100	0	98	18	Unknown	3	0	0	1	0
18	60	Male	II	Lung	Thoracic vertebra	Debulking	700	400	201	32	28	0	1	3	1	0
19	66	Male	II	Lung	Thoracic vertebra	Decompre-ssion	400	400	169	8	4	0	8	1	21	0
20	63	Female	II	Lung	Thoracic vertebra	Decompre-ssion	200	0	166	2	Unknown	0	1	1	2	1

S1: number of CTCs / 4 ml in S1; S2: number of CTCs / 4 ml in S2; S3: number of CTCs / 4 ml in S3; S4: number of CTCs / 4 ml in S4

### Medical history of the primary tumour

**Fig. 2:**
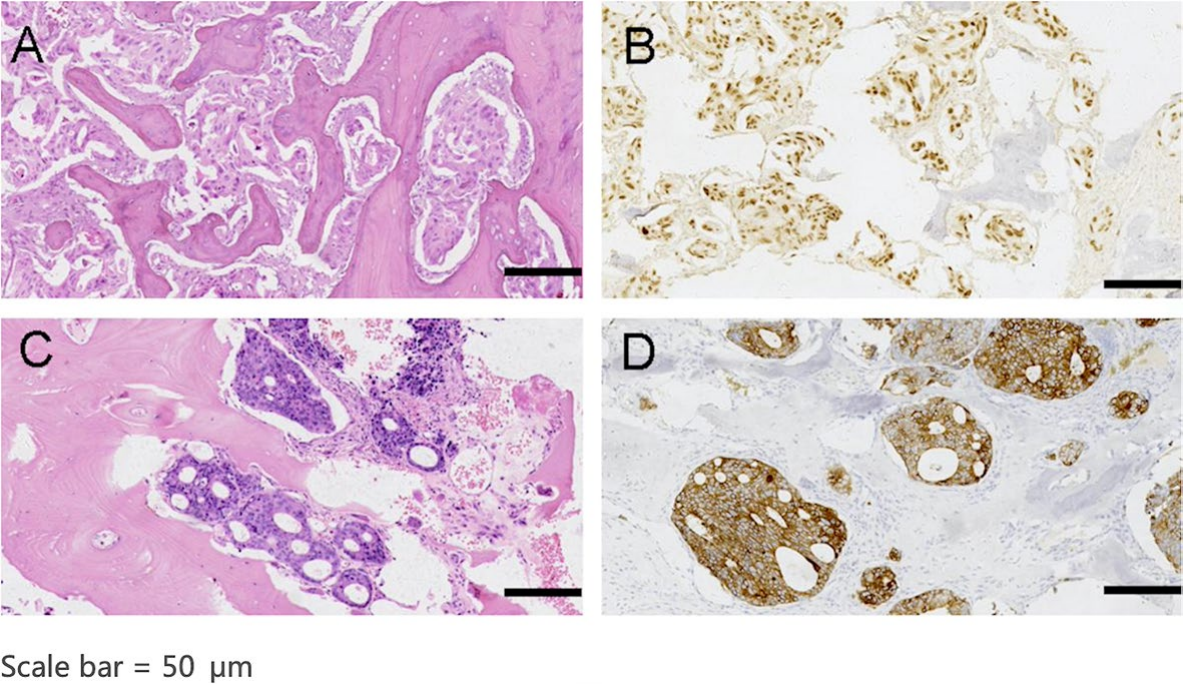
The pathology images of MSTS. **A** Bone metastasis of breast cancer stained with hematoxylin-eosin staining (HE); **B** Bone metastasis of breast cancer stained with GATA3; **C** Bone metastasis of colon cancer stained with HE; **D** Bone metastasis of colon cancer stained with CK20. Scale bar = 50 μm

The origin of the primary lesions was mainly in the lungs (6 cases), gastrointestinal tract (6 cases, including 2 in the colon, 2 in the oesophagus, 1 in the rectum, and 1 in the ampulla), breasts (3 cases), thyroid (2 cases), endometrium (1 case), nasopharynx (1 case), and renal (1 case) (Table 2), which were confirmed by pathological evaluation of paraffin sections stained with haematoxylin-eosin and by immunohistochemistry (Fig. 2). Of the 20 patients recruited, 7 underwent primary tumour resection, 9 had a history of chemotherapy, 1 had a history of targeted therapy, 2 had a history of radiotherapy, all for primary tumours. All radiotherapy and chemotherapy had little impact on the number of tumour cells in the blood samples (Table 3).

**Table 2:** Primary tumour origin and vertebral levels involved

Primary tumour	vertebral levels involved
Lung - 6	Cervical vertebra - 5
Gastrointestinal - 6	Cervicothoracic vertebra - 1
Colon - 2	Thoracic vertebra - 10
Esophagus - 2	Lumbar vertebra - 4
Rectum - 1	
Ampulla - 1	
Breast - 3	
Thyroid - 2	
Renal - 1	
Endometrium - 1	
Nasopharynx - 1

**Table 3:** Medical history of the primary tumour

History of treatment		No. of patients	No. of patients of S1 = 0(%)	No. of patients of S2 = 0(%)	No. of patients of S3 = 0(%)	No. of patients of S4 = 0(%)
chemotherapy	Yes	9	1 (11.1)	2 (22.2)	3 (33.3)	3 (33.3)
	No	11	5 (45.5)	2 (18.2)	4 (36.4)	5 (45.5)
Targeted therapy	Yes	1	0	0	0	0
	No	19	6 (31.6)	4 (21.1)	7 (36.8)	8 (42.1)
Radiotherapy	Yes	2	0	0	0	0
	No	18	6 (33.3)	4 (22.2)	7 (38.9)	8 (44.4)
Primary lesion resection	Yes	7	1 (14.2)	0	2 (28.6)	2 (28.6)
	No	13	5 (38.5)	4 (30.8)	5 (38.5)	6 (46.2)

### Detection of tumour cells

**Fig. 3:**
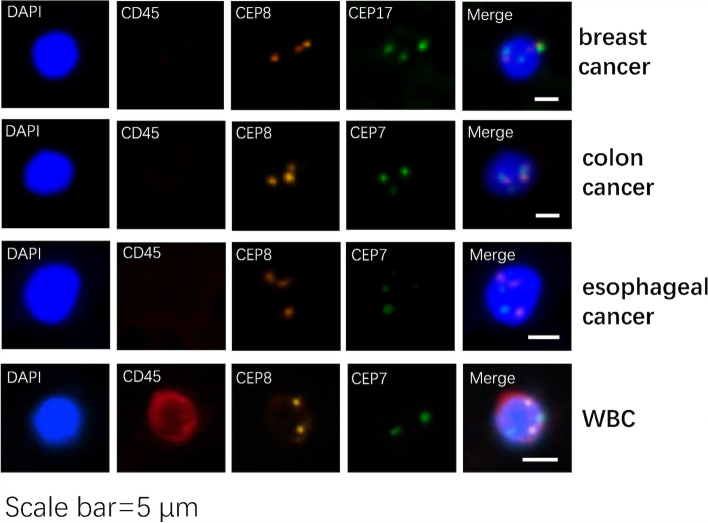
Detection of tumour cells. The expression level of CD45 and the number of chromosome, such as CEP 8, CEP 17 or CEP 7 could be distinguished by immunofluorescence staining and FISH. The cells with negative CD45 expression and one or all of CEP 8, CEP 17 or CEP 7 abnormal number (number ≥ 3) were identified as circulating tumour cells. The positive expression of CD45 were discriminated as WBC. CEP 8 is the centromere signal of chromosome 8, CEP 7 is the centromere signal of chromosome 7, CEP 17 is the centromere signal of chromosome 17. Scale bar: 5 μm

Overall, 88 samples from 20 patients (including 20 samples each for S1, S2, S3, and S4 and 8 samples for S5) were analysed. After excluding CD45+ leucocytes using immunomagnetic beads (Fig. 3), aneuploidy (abnormal chromosome numbers) was detected in CEP 8, CEP 17, or CEP 7 by FISH. Tumour cells were recognized and counted using immunofluorescence staining and FISH. Negative expression of CD45 and aneuploidy of CEP 8, CEP 17, and CEP 7 were considered to indicate malignant cells (Fig. 3).

### Ability of MLDF and RLDF to eliminate tumour cells

**Fig. 4:**
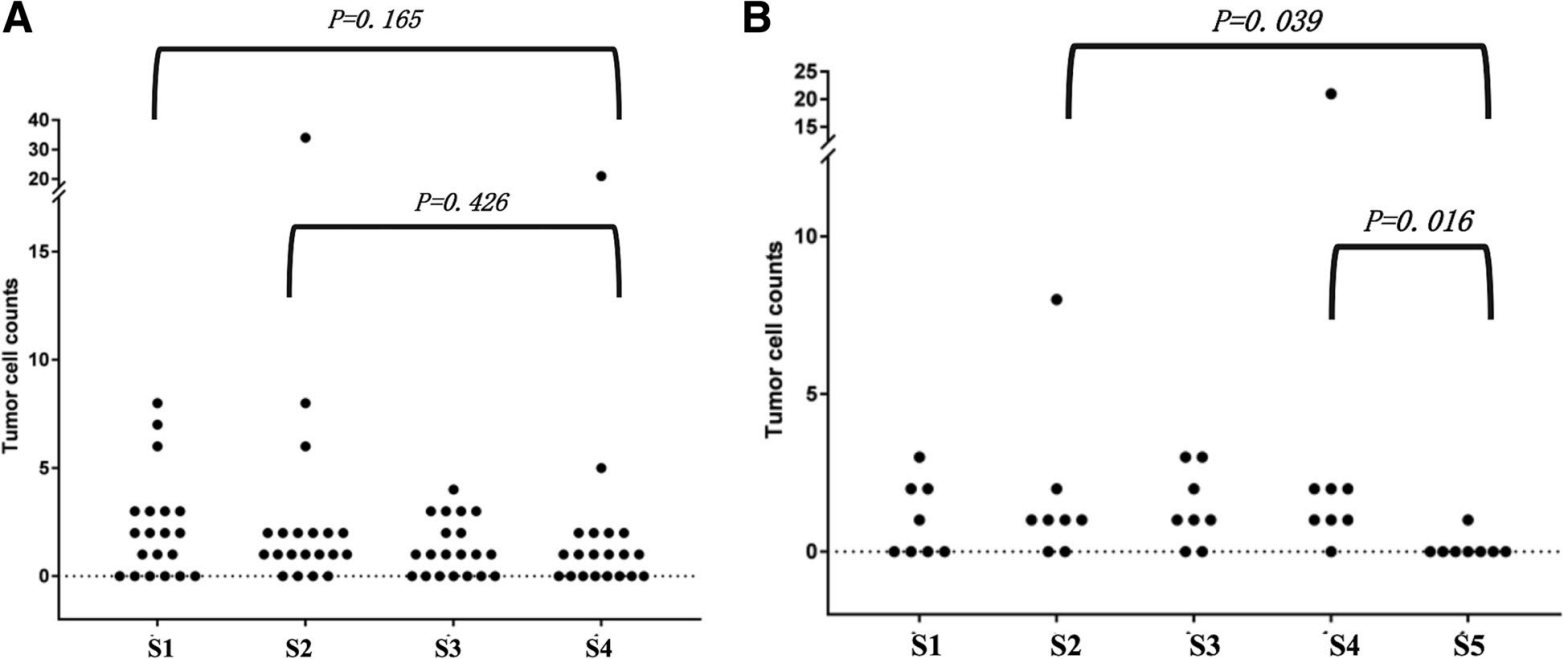
All of tumour cell counts in each sample (4 ml). **A** Tumour cell counts in 20 cases processed by RLDF; **B** Tumour cell counts in 8 cases processed by RLDF and MLDF

The number of tumour cells in all of the samples is shown in Table 1 and Fig. 4. Tumour cells were detected in S1 in 14 patients (70%), S2 in 16 patients (80%), S3 in 13 patients (65%), and S4 in 12 patients (60%) (Table 1 and Fig. 4A). In 8 patients, MLDF was used, and tumour cells were detected in only 1 patient (12.5%) in S5 (Table 1 and Fig. 4B). There was no significant difference between S4 and S1 (*P* = 0.165) or between S4 and S2 (*P* = 0.426), although a downward trend appeared after IOCS+RLDF processing. The number of tumour cells was significantly lower in the S5 samples after MLDF processing than in the S4 and S2 samples (*P* = 0.016 and *P* = 0.039, respectively). The number of tumour cells was not significantly increased in S5 compared to S1 (*P* = 0.102).

## Discussion

Blood loss during MSTS remains challenging, although minimally invasive surgical techniques have greatly improved. Blood loss varies greatly depending on the primary tumour of spinal metastases, surgical approaches, and operative time [2]. Recently, Kumar et al. found that the mean bleeding volume was 870 ± 720 mL, and the average blood transfusion volume was 1.5 ± 1.9 U in MSTS [2]. Even in minimally invasive spinal metastatic tumour surgery, the average bleeding volume has been reported as 745 mL (184–1320 mL) [15]. In the present study, the average volume of blood loss was 392.5 ± 270.61 mL, which is less than that reported in other studies. Eleven patients received ABT with an average transfusion volume of 280 ± 293.08 mL. The prevalence of anaemia in patients with cancer is reported to be approximately 40% [16] due to nutritional deficiency, chronic disease, blunted response to erythropoietin, bone marrow suppression either due to cancerous cells or as a side effect of chemotherapy/radiotherapy, and other causes [17]. Transfusion requirements are usually larger than expected. Thus, the investigation of IOCS+LDF is valuable for patients.

There is still controversy around the clinical safety of using salvage blood in oncological surgeries, despite literature establishing its safety. LDF is a filter device based on a membrane-like filter material used to remove leucocytes from the blood. The mechanisms underlying the removal of tumour cells are physical interception and charge adsorption based on cell size. The size of white blood cells is approximately 7–20 μm. The RLDF refers to a white blood cell filter with a pore size of 40 μm. Gray et al. conducted a prospective cohort study and found no significant difference in progression-free survival between preoperative autologous donation and IOCS-LDF groups undergoing prostatectomy [18]. Patients who undergo partial hepatectomy for colorectal cancer metastases can safely receive a transfusion of filtered autologous blood, which is not associated with an increased risk of recurrence or a higher mortality rate [19]. Patients who received a salvaged blood transfusion required significantly lesser amounts of allogeneic blood, and their survival rates and disease progression remained lower or similar to that in control patients. Furthermore, there are many studies on the clinical safety of salvage blood used in oncological surgeries, including gynaecological [20], hepatobiliary [21, 22], gastrointestinal [23], urological [18], and pulmonary [24] surgery. However, there are only a few studies on IOCS+LDF reinfusion in patients with MSTS. Gakhar et al. observed that transfusion of intraoperatively salvaged blood did not result in disseminated metastatic cancer in MSTS (level of evidence IV) [25]. More recently, Elmalky et al. demonstrated that the use of IOCS-LDF in MSTS reduces the need for postoperative ABT while maintaining satisfactory postoperative haemoglobin and they recommended routine use of IOCS-LDF in MSTS for its safety, efficacy, and potential cost benefit [4].

In this study with 20 patients, tumour cells were detected in the blood samples of 12 patients (60%) after IOCS+RLDF processing (S4), whereas it was found in venous blood before surgery (S1) in 14 (70%), in blood from the operative field (S2) in 16 (80%), and in blood after IOCS (S3) in 13 patients (65%). There was no significant difference between S4 and S1 or between S4 and S2, although a downward trend appeared after IOCS+RLDF processing (Fig. 4). This may imply that RLDF could not completely eliminate tumour cells. The “RLDF” we used did not seem to perform well compared with other published literature on IOCS with LDF in MSTS [6, 7, 26]. Our study employed the same inclusion criteria as Professor Kumar’s team, all of which were patients with a known primary epithelial tumour who underwent MSTS. In our study, leukocytes were first removed by magnetic bead sorting, and FISH technology was then used to identify chromosomal polyploidy. Different detection methods may have an impact on tumour cell recognition, resulting in poor RLDF results in our study. Consequently, new filters were added into the experiment, and got relatively satisfactory results.

Studies indicate that the pore size of the filter membrane and the wash solution are two key elements for cell depletion. Considering metastatic spine tumour derived from various types of primary tumours, the tumour cells in the blood are of different sizes. The new filter with smaller pore size was added into the experiment, and got relatively satisfactory results. MLDF was developed by a medical team in Sichuan, China. It is used for research purposes only at present and has not been commercially available. Mei et al. reported a 3–4 log reduction in leucocytes using MLDF with a pore size of 12–18 μm [8]. RLDF-treated samples were subsequently inoculated in nude mice, 67% of which developed tumours. In contrast, no tumour cells were found in MLDF-treated samples, and no solid tumours were observed in inoculated nude mice [8]. Therefore, it is considered that MLDF with mannitol-adenine-phosphate solution had higher filter efficiency, but further clinical research is warranted. Thus, we explored MLDF with a pore size of 18 μm to filter spinal metastasis tumour cells from known primary epithelial tumours. The number of tumour cells was significantly lower in the samples after MLDF processing (S5) than in the operative field (S2). We also confirmed that MLDF was more effective in eliminating tumour cells than RLDF. In this study, MLDF was more efficient for filtering tumour cells, which suggests that it may have great prospects for managing blood salvage in oncologic surgery.

Ideally, we try to eliminate all tumour cells from salvaged blood. In our study, IOCS+RLDF reduced the number of tumour cells in patients’ peripheral venous blood, but it did not eliminate them completely. MLDF achieved a much better result, clearing nearly all tumour cells except in one case (No. 20). The patient with a primary lung cancer had the shortest survival time (2 months) and had a pathological type of low-differentiated squamous cell carcinoma, possibly suggesting that tumour cells with a high degree of malignancy can easily escape from LDF. Currently there is laboratory evidence that MLDF-filtered samples do not cause tumours in mice [8], but no evidence of clinical studies has been found. In the future, we will continue to explore the filtering effect of IOCS and LDF on MSTS from different pathological types, and hopefully propose clinical evidences for the safety of using IOCS. Karczewski et al. demonstrated that 62% of the tumour cells in blood underwent lethal trauma, whereas all the remaining tumour cells displayed morphological changes, after being processed with the IOCS device [27]. Kumar et al. reported that the tumour cells that pass through the IOCS device are morphologically altered and become nonviable and that they lose their ability to form new metastatic deposits [28]. Similar results were observed in other studies [7, 20]. Therefore, we assume that IOCS+MLDF would be an effective strategy to destroy and eliminate malignant cells and that salvaged autologous blood can be safely reinfused to the patient.

### Strength and limitations of this study

- MLDF was applied for the first time on patients of MSTS in China.
- The salvaged blood used in this study was not reinfused to patients because of the limitations imposed by ethical issues.
- We failed to clarify the viability and functionality of the tumour cells because there were too few residual tumour cells to evaluate. And this will be a focus of our future work.

## Conclusions

This study evaluated the effectiveness of IOCS combined with MLDF in MSTS. Although tumour cells could be removed by IOCS combined with RLDF from blood salvaged during MSTS, there were still residual tumour cells remaining. These findings support the notion that MLDF eliminates tumour cells more effectively than RLDF. Hence, this technique can be applied in MSTS.

## Data Availability

The data during this study are available from the corresponding author on reasonable request.

## Abbreviations

IOCS: Intraoperative cell salvage
MLDF: Modified leucocyte depletion filter
RLDF: Regular leucocyte depletion filter
MSTS: Metastatic spine tumour surgery
ABT: Allogeneic blood transfusion
LDF: Leucocyte depletion filter
FISH: Fluorescence in situ hybridization
CEP: Chromosome enumeration probe
HE: Haematoxylin-eosin
FISH: Fluorescence in situ hybridisation
